# American College of Dental Surgery, Chicago

**Published:** 1892-09

**Authors:** 


### AMERICAN COLLEGE OF DENTAL SURGERY, CHICAGO.

It is a pleasure to be able to announce that this College has been reinstated in the National Association of Dental Faculties.

Since its suspension, a year ago, it has been thoroughly reorganized, with a Faculty composed of men able to give it a high standing in Chicago. The action taken with this school was a valuable object-lesson, in that it demonstrated that no college could openly violate a rule established for the good of the entire body without suffering the penalty. The suspension or expulsion of a college from this organization means its ruin as an educational institution.

